# Genome-Wide Survey Reveals the Microsatellite Characteristics and Phylogenetic Relationships of *Harpadon nehereus*

**DOI:** 10.3390/cimb43030091

**Published:** 2021-09-25

**Authors:** Tianyan Yang, Xinxin Huang, Zijun Ning, Tianxiang Gao

**Affiliations:** Fisheries College, Zhejiang Ocean University, Zhoushan 316022, China; yangty@zjou.edu.cn (T.Y.); 18281060997@163.com (X.H.); Ningzijun2020@163.com (Z.N.)

**Keywords:** *Harpadon nehereus*, genome survey, microsatellite identification, phylogenetics

## Abstract

*Harpadon nehereus* forms one of the most important commercial fisheries along the Bay of Bengal and the southeast coast of China. In this study, the genome-wide survey dataset first produced using next-generation sequencing (NGS) was used to provide general information on the genome size, heterozygosity and repeat sequence ratio of *H. nehereus*. About 68.74 GB of high-quality sequence data were obtained in total and the genome size was estimated to be 1315 Mb with the 17-mer frequency distribution. The sequence repeat ratio and heterozygosity were calculated to be 52.49% and 0.67%, respectively. A total of 1,027,651 microsatellite motifs were identified and dinucleotide repeat was the most dominant simple sequence repeat (SSR) motif with a frequency of 54.35%. As a by-product of whole genome sequencing, the mitochondrial genome is a powerful tool to investigate the evolutionary relationships between *H. nehereus* and its relatives. The maximum likelihood (ML) phylogenetic tree was constructed according to the concatenated matrix of amino acids translated from the 13 protein-coding genes (PCGs). Monophyly of two species of the genus *Harpadon* was revealed in the present study and they formed a monophyletic clade with *Saurida* with a high bootstrap value of 100%. The results would help to push back the frontiers of genomics and open the doors of molecular diversity as well as conservation genetics studies on this species.

## 1. Introduction

*Harpadonnehereus* (Hamilton, 1822), also known as Bombay duck or nomei fish, is generally distributed in the estuarine and nearshore shallow waters of the Indian Ocean and the Western Pacific Ocean [1]. As a familiar marine lizardfish, *H. nehereus* is welcomed by consumers for its delicious taste and high nutritional value. It is admeasured that the protein content reaches up to 70% of the dry weight and the calcium content ranges from 1500 to 2500 mg/100 g [2]. Nowadays, it is one of the most important commercial fisheries along the coast of southeast China, Pakistan, India and the Bay of Bengal region [3,4,5,6]. In China, the potential amount of *H. nehereus* in the East China Sea was estimated to surpass 5000 tons, which indicated that *H. nehereus* occupied a significant position in the coastal marine ecosystem of China. Nevertheless, in recent years, a recession of *H. nehereus* resources has emerged along with the increasing proportion of annual capture yield [6,7,8]. Therefore, it is necessary to strengthen protection of this fishery in a sustainable way, and further ensure rational exploitation and continued utilization of *H. nehereus* resources.

Investigation of genetic background is an essential precondition for fishery resource management [9]. However, most studies on *H. nehereus* have been conducted focusing on stock assessment, morphological traits, biological habits and nutrition processing [10,11,12,13], and limited studies involve the field of genetics. For example, these studies include population genetic structure and genetic variation analysis based on simple sequence repeat (SSR), sequence-related amplified polymorphism (SRAP) and mitochondrial DNA (mtDNA) markers [14,15,16,17], the molecular phylogenetic relationship revealed by mtDNA Cyt b, 16S rRNA and the complete mtDNA genome [17,18,19], and the expression analysis of calcium cycling genes by transcriptome sequencing [20]. With the accomplishment of the Human Genome Project (HGP) early in 2001, a new era of genomics has arrived in the field of genetics research. Thus far, more and more genome sequencings of economic fishes such as *Larimichthys crocea*, *Cynoglossus semilaevis*, *Salmo salar*, *Oncorhynchus mykiss* and *Gadus morhua* have been carried out along with the rapid development of high-throughput sequencing technology [21]. However, the genomic resource for *H. nehereus* is still extremely scarce and its full draft genome remains unclear. Therefore, in this study, we conducted a genomic survey of *H. nehereus* via next-generation sequencing (NGS) technology for the first time to investigate its genomic profile, excavate the SSR markers and further identify the taxonomic position and phylogenetic relationship of *H. nehereus*. The results might provide new insight into genetic resource conservation and developmental utilization for *H. nehereus.*

## 2. Materials and Methods

### 2.1. Sample Collecting

A specimen of a male *H. nehereus* (body length 16.1 cm and body weight 26.9 g) was obtained from coastal waters by a trawl net in autumn 2020. Approximately 2–3 g fresh musculature below the dorsal fin was snipped and soaked in liquid nitrogen as soon as the fish was captured. The sample was immediately brought to the Fisheries Ecology and Biodiversity Laboratory (FEBL) of Zhejiang Ocean University and stored in a −80 °C ultra-low temperature freezer. All animal experiments were conducted according to the guideline and approval of the Ethics Committee for Animal Experimentation of Zhejiang Ocean University, Zhoushan, China. The project identification code was ZJOU-ECAE20210128 and approval date was 6 January 2021.

### 2.2. DNA Extraction, Library Construction and Illumina Sequencing

The genomic DNA was isolated using a standard phenol–chloroform method [22]. The integrity of DNA was examined by 1% agarose gel electrophoresis. The quality of DNA was assessed using a NanoPhotometer spectrophotometer (Implen, Munich, Germany), Qubit 2.0 fluorometer (Invitrogen, Waltham, MA, USA) and Agilent 2100 bioanalyzer (Agilent, Santa Clara, CA, USA). The qualified DNA sample was randomly interrupted by an ultrasonic crusher (Covaris M220, Woburn, MA, USA), and the DNA fragments (300–350 bp) were used to construct two paired-end DNA libraries followed by terminal repair, adding A-tail and sequencing adaptors, purification and PCR amplification. The constructed library was then sequenced by Wuhan Gooalgene Technology Co., Ltd., Wuhan, China (https://www.gooalgene.com/) (accessed on 18 March 2021) based on the Illumina Nova platform with a read length of 2 × 150 bp. The sequencing data were deposited in the short-read archive (SRA) database (http://www.ncbi.nlm.nih.gov/sra/) (accessed on 15 June 2021) under the accession number PRJNA738314.

### 2.3. Sequence Quality Control, Assembly and K-mer Analysis

Raw data were first converted to single-sample FASTQ files through base calling and then filtered by SOAPnuke v1.5 [23] with the following criteria: (1) removing reads with splice junctions, (2) discarding duplicated reads caused by PCR reaction or other reasons, (3) deleting reads with N base (unable to determine base information) ratio greater than 10% and low-quality bases (base quality ≤ 5) more than 50% of the total length. Clean data were de novo assembled into contigs and scaffolds by SOAPdenovo v2.01 software [24]. The K-mer analysis was performed to estimate genome size, heterozygosity and repetitive sequences of the genome using Jellyfish v2.2.10 (http://www.genome.umd.edu/jellyfish.html) (accessed on 9 April 2021) and a de Bruijn graph assembler GenomeScope (http://qb.cshl.edu/genomescope/) (accessed on 21 April 2021). Genome size could be expressed as the formula: K-mer number/peak depth [25]. Non-overlapping sliding windows of 10 kb along the assembled sequence were used to calculate the GC content and average depth [26,27].

### 2.4. Microsatellite Identification and Phylogenetic Analysis

Perl script MISA (MIcroSAtellite identification tool) was used to identify simple sequence repeats (SSRs) in the genome of *H. nehereus* [28]. The parameters were set for the detection of di-, tri-, tetra-, penta- and hexanucleotide SSR motifs with a minimum of 6, 5, 5, 5 and 5 repeats, respectively. The distribution and frequency of SSRs were computed and mapped by Microsoft Excel. The complete mitochondrial DNA (mtDNA) sequence of *H. nehereus* was assembled by MITObim version 1.9.1 (https://github.com/chrishah/MITObim) (accessed on 20 May 2021) and annotated using the online tool MITOS (http://mitos2.bioinf.uni-leipzig.de/index.py) (accessed on 26 May 2021). The mtDNA sequence was corrected manually comparing with published sequences of *H. nehereus* (GenBank accession numbers: JX534239 and MH204885). The nucleotide composition and genetic relationships were analyzed by MEGA 11 [29]. Phylogenetic analysis was performed using fifty-eight complete mtDNA genomes of related species downloaded from GenBank, with *Ijimaia dofleini* (GenBank accession number: AP002917) selected as an outgroup. The maximum likelihood (ML) method was used to construct the phylogenetic tree based on concatenated amino acids of the 13 protein-coding genes (PCGs).

## 3. Results

### 3.1. Sequencing Data Statistics and Sequence Quality Evaluation

**Figure 1:**
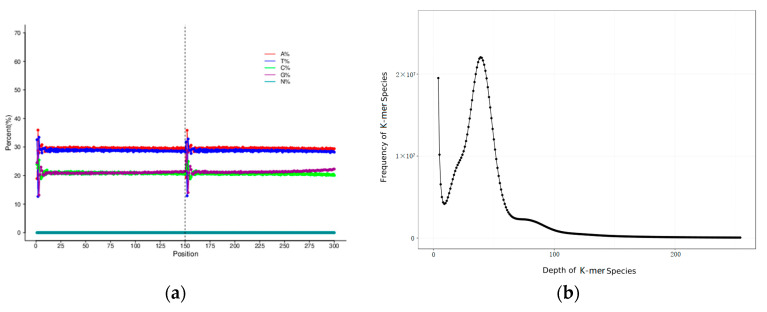
The base distribution and K-mer (K = 17) analysis of *H. nehereus*. (**a**) On the *x*-axis, the read-1 base contents are on the left side of the dotted line and the read-2 base contents are on the right. Different colors represent different base types. The *y*-axis represents the sequencing depth. (**b**) The *x*-axis means K-mer depth and the *y*-axis represents the frequency for the corresponding depth.

After sequence filtering and correction, a total of 68.74 Gb high-quality data were generated by the Illumina Nova platform with high-throughput paired-end sequencing in this study. The Q20 (base quality > 20) value, Q30 (base quality > 30) value and GC content were 96.64%, 91.35% and 41.78%, respectively, with the approximate sequencing depth of 50×. Ten thousand pairs of reads were randomly selected from the filtered high-quality data and compared with the Nucleotide Sequence Database (NT) from the National Center for Biotechnology Information (NCBI) database using the Basic Local Alignment Search Tool (BLAST). The results showed the library was successfully compared to the related species of *H. nehereus*, which proved that there was no obvious exogenous contamination during the library construction. The proportion of single bases presented the separation of AT content and GC content. In addition, the N content was close to zero (Figure 1a). All of this suggested that the sequencing quality was good.

### 3.2. Genome Assembly, Heterozygosity and Repeat Prediction

The de Bruijn graph-based assembler SOAPdenovo was applied to generate a total length of 1.35 Gb contigs with an N50 length of 596 bp. A total number of 2,539,084 sequences (the max length was 92,118 bp) comprised approximately 1.36 Gb scaffolds with an N50 length of 1568 kb (Table 1). K-mer analysis (K = 17) was used to estimate the genomic characteristics. The K-mer distribution map is shown in Figure 1b. The K-mer number obtained from the sequencing data was 54,514,460,352, with a K-mer depth of 39. The revised genome size of the diploid fish *H. nehereus* was 1315 Mb after eliminating the effects of erroneous K-mers. The heterozygosity ratio and repeat sequence ratio were 0.67% and 52.49%, respectively.

### 3.3. The Distribution and Characteristics of SSR Loci

**Figure 2:**
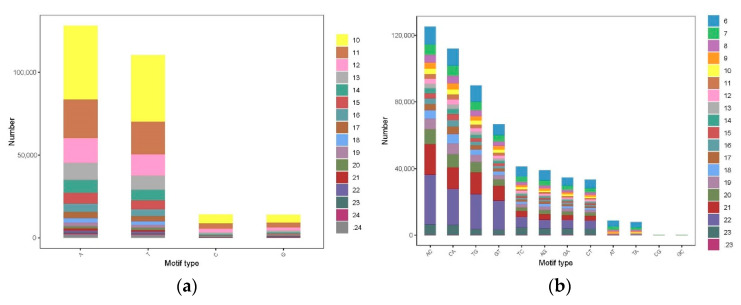

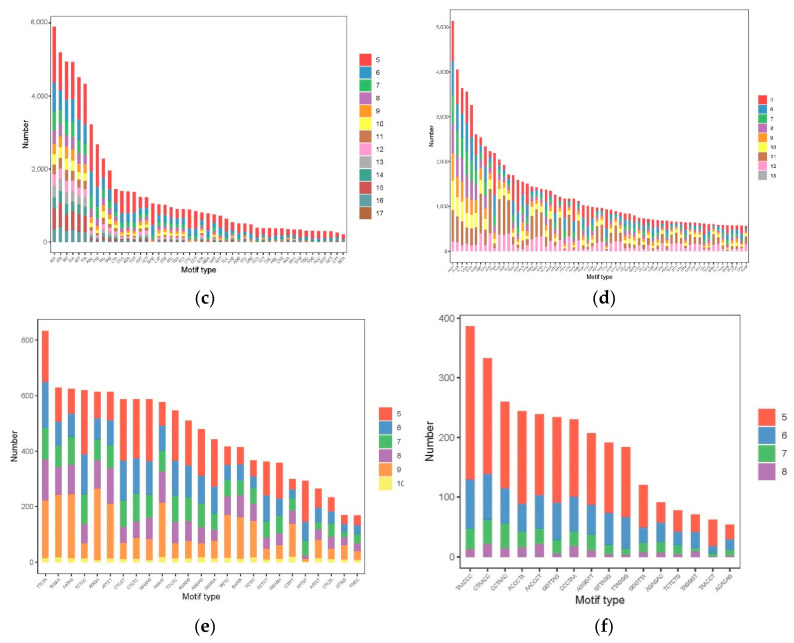
The distribution and frequency of microsatellite motifs in *H. nehereus*. (**a**–**f**) present frequency of the mono-, di-, tri-, tetra-, penta- and hexanucleotide microsatellite motifs.

A total of 1,027,651 SSR-containing motif repeats were identified, with a total SSR length of 27,355,367 bp (Table 2). Among all these SSRs, the mono-, di-, tri- and tetra-nucleotide repeats contributed nearly 99% of SSRs in *H. nehereus*. The dinucleotide repeat was the most abundant SSR marker, accounting for 54.35% of the total SSR markers, which was followed by mononucleotide (25.96%) and tetranucleotide (10.26%) repeats. The percentage of hexaucleotide repeats was the lowest of all (0.53%). The frequency of different repeat motifs is presented in Figure 2. The frequency of A/T repeats was the highest within the four types of mononucleotide repeat. Among the dinucleotide microsatellite motifs, the CA/TG repeat motif was the most abundant, which accounted for 19.64%, followed by AC/GT at 18.66%. The AAT/ATT (1.05%) repeat motif was the most frequent among all types of repeats. Additionally, among the tetranucleotide repeat motifs, the common motifs were AGAC/GTCT and ACAG/CTGT, accounting for 0.63% and 0.71%, respectively.

### 3.4. Mitochondrial DNA Assembly and Phylogenetic Relationships of H. nehereus

**Figure 3:**
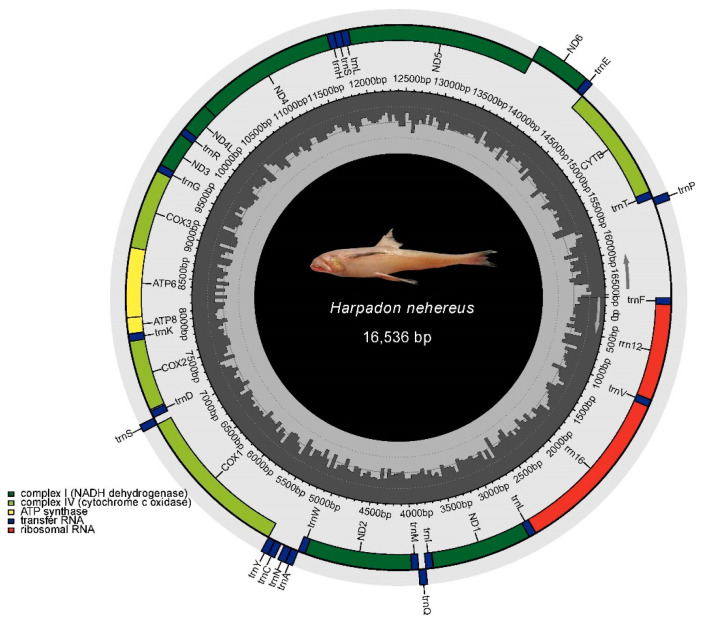
The complete mitogenome structure of *H. nehereus*.

**Figure 4:**
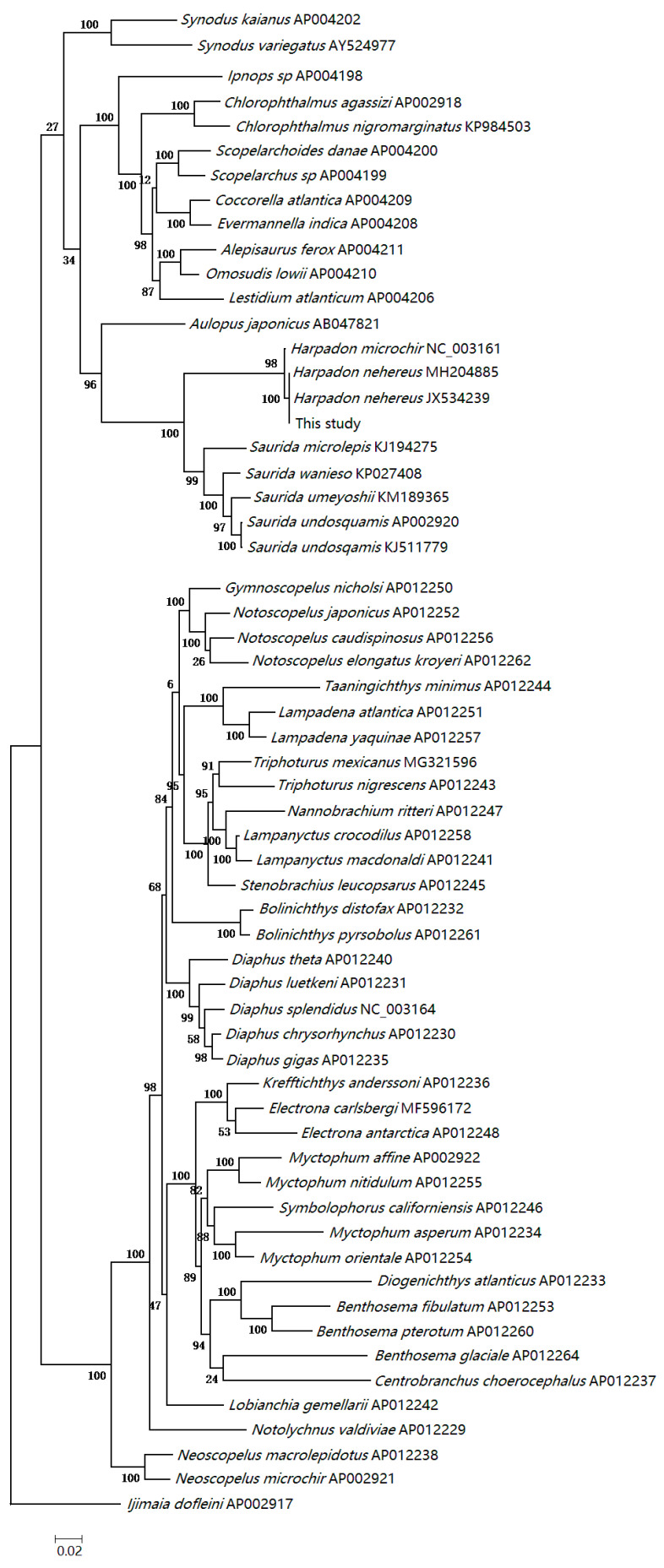
The phylogenetic tree inferred from mitochondrial genomes of related species.

The total length of the complete mitogenome of *H. nehereus* was 16,536 bp. Just like other published bony fishes’ mtDNA genomes [30], the closed circular molecule included the 13 PCGs, two ribosomal RNA genes, 22 transfer RNA genes and a control region (Figure 3). Most mitochondrial genes were encoded on the H-strand, excluding ND6 and eight tRNAs (Gln, Ala, Asn, Cys, Tyr, Ser-UCN, Glu and Pro) that were located on the L-strand. The nucleotide percentages were as follows: A = 26.75%, T = 26.74%, G = 16.73% and C = 29.78%. The proportion of A + T (53.49%) was higher than that of G + C (46.51%), indicating an AT bias. The phylogenetic tree was constructed using the ML method based on combined amino acids translated from the 13 PCGs (Figure 4). The topological structure showed that all individuals were divided into two clades. Species of the families Myctophidae and Neoscopelidae formed one clade, and the rest were clustered into the other one. *H. nehereus* and *H. microchir* were the closest relatives, and they clustered with the genus *Saurida*. Members of the suborder Alepisauroidei gathered together and had close relationships with *Chlorophthalmus agassizi*, *C. nigromarginatus* and *Ipnops* sp.

## 4. Discussion

The rapid development of high-throughput sequencing technology not only greatly reduces the sequencing cost, but also significantly shortens the sequencing cycle and promotes the research process of whole genome sequencing (WGS) and genetic mapping [31,32]. Since the first fish genome (known as “torafugu” *Fugu rubripes*) was published in 2002 [33], WGS has been explosively applied into more and more fish species, ranging from the model fish medaka [34] and zebrafish [35] to many commercially important fishes. Particularly in recent years, the implementation of “The China Aquatic 10-100-1000 Genomics Program” and the “Fish 10K Project” have brought the sequencing of fish genome into a new stage [36,37]. Genomic approaches have shown their great powers in conservation and utilization of fishery resources, exploration of species evolution, molecular breeding, research and development of marine drugs and disease control [38,39]. In this study, we reported the genome survey of an important economic lizardfish, Bombay duck, using the WGS method for the first time. In addition, the microsatellite and mtDNA markers were also identified and characterized for genetic diversity and population structure studies in the future.

According to the K-mer analysis, the genome size of *H. nehereus* was estimated to be 1315 Mb, which was smaller than the predicted genome size based on the DNA content [40]. This possibly resulted from the non-specific fluorescent dye binding to non-genomic nucleotides. Until now, the published teleost genome size has ranged from 322.5 Mb (*Fugu rubripes*) [33] to 40 Gb (*Protopterus annectens*) [41]. Most of the commercial marine species have a genome size of less than 1 Gb, such as *Larimichthys crocea* [42], *Paralichthys olivaceus* [43], *Lateolabrax maculatus* [44] and *Thamnaconus septentrionalis* [45]. Additionally, compared with *Benthosema glaciale* (accession number: PRJEB12469), the only reported genome of lanternfishes, it was almost twice as much as that of this species (approximately 676 Mb). It was inferred that the genome size of *H. nehereus* was relatively large, as a result of the higher repetitive sequence ratio (52.49%). Sequence bias would occur in Illumina sequencing libraries if the GC content was out of the range of 25–65%, and thus seriously affect genome assembly [46]. The GC content of the *H. nehereus* genome was 41.78%, which was within the acceptable range for genome assembly.

Heterozygosity is another important factor affecting genome assembly and subsequent analysis. As both sister chromatids would be separately assembled in high-heterozygosity regions, it might cause the imprecise assessment of genome size. The heterozygosity ratio of *H. nehereus* observed in the present study was 0.67%, which was higher than that of *Scatophagus argus* (female 0.37% and male 0.38%) [47], *Sebastiscus marmoratus* (0.17%) [48] and *Acanthogobius ommaturus* (0.17%) [49], but smaller than that of *Sillago sihama* (0.92%) [50]. Genome assembly becomes more difficult when the heterozygosity ratio is higher than 0.5% [25]. Therefore, the higher heterozygosity ratio might impact the accuracy of genome size estimation. 

As a powerful and popular tool to unravel the higher-level relationships of teleosts, complete mtDNA has been more and more widely applied in the studies of evolutionary biology and phylogenetics [30,51]. The genome-wide data include both nuclear DNA information and mitochondrial DNA sequences, hence we excavated the complete mitogenome of *H. nehereus* to further decipher its taxonomic status and systematic evolution. Rosen discovered the distinctive features of the second and third pharyngobranchials in Aulopiformes fishes, and separated them from Myctophiformes for the first time [52]. Subsequently, Nelson agreed with Rosen’s taxonomy in the reprinted “*Fishes of the World* (2nd Edition)” on the basis of morphological structures of gill rakers, and he regarded the Myctophiformes (Myctophidae + Neoscopelidae) and Aulopiformes (Auiopoidei + Alepisauroidei) as two different orders [53]. In our study, the cladogram constructed using a concatenated set of amino acid sequences from the 13 PCGs supported the above-mentioned taxonomical criteria, and revealed a monophyly of Myctophiformes that was corroborated by morphological characteristics and molecular evidence [54,55,56,57]. The evolutionary tree also showed that *H. nehereus* and *H. microchir* belong to the family Synodontidae located in the same clade of Aulopiformes and had the closest relationships with each other. The genus *Harpadon* was inferred as a sister group to *Saurida* with a strong bootstrap value, which was consistent with the results of a previous study using 16S rRNA and COI genes [19,58].

## 5. Conclusions

This was the first report of the genome survey analysis of a commercially important marine lizardfish (Bombay duck) based on the Illumina platform. The genome size of *H. nehereus* was 1315 Mb, with 2,539,084 scaffolds and an N50 length of 1568 bp. The candidate SSR and mitochondrial markers developed from *H. nehereus* genome data could provide novel insight into population genetics and germplasm resource conservation in the future. Considering that the higher level of repetitive DNA and heterozygosity might make it more challenging to generate highly accurate de novo genome assembly, the strategy of Illumina combined with PacBio and Hi-C techniques should be applied to obtain a higher-quality genome of *H. nehereus*, and more efforts are still to be made in the further research on the genome of this species.

## Figures and Tables

**Table 1:** Assembly statistics for stitched contigs and scaffolds of *H. nehereus*.

Sample		Total Length (bp)	Total Number	Total Number (≥2 kb)	Max Length (bp)	N50 (bp)	N90 (bp)
*H. nehereus*	contigs	1,352,668,147	3,766,078	73,281	30,284	596	138
	scaffolds	1,363,443,545	2,539,084	133,089	92,118	1568	162

**Table 2:** The statistics of SSRs in the *H. nehereus* genome based on repeat types.

SSR Type	Number	Percent (%)	Sequence Number	Total SSR Length (bp)
p1	266,761	25.96	208,219	3,369,363
p2	558,543	54.35	400,985	17,745,246
p3	66,270	6.45	62,046	1,638,864
p4	105,426	10.26	97,016	3,527,488
p5	25,182	2.45	23,958	883,000
p6	5469	0.53	5374	191,406
Total	1,027,651	100	797,598	27,355,367

## Data Availability

All data presented during this study are included in this article.
